# Artificial Intelligence (AI)-Based Detection of Anaemia Using the Clinical Appearance of the Gingiva

**DOI:** 10.7759/cureus.62792

**Published:** 2024-06-20

**Authors:** Shubhangini Chatterjee, Sankari Malaiappan, Pradeep Kumar Yadalam

**Affiliations:** 1 Periodontics, Saveetha Dental College and Hospitals, Saveetha Institute of Medical and Technical Sciences, Saveetha University, Chennai, IND

**Keywords:** dental, gingiva, automatic clinical diagnosis, innovative, deep learning, artificial intelligence, anaemia

## Abstract

Background and aim

Millions suffer from anaemia worldwide, and systemic disorders like anaemia harm oral health. Anaemia is linked to periodontitis as certain inflammatory cytokines produced during periodontal inflammation can depress erythropoietin production leading to the development of anemia. Thus, detecting and treating it is crucial to tooth health. Hence, this study aimed to evaluate three different machine-learning approaches for the automated detection of anaemia using clinical intraoral pictures of a patient’s gingiva.

Methodology

Orange was employed with squeeze net embedding models for machine learning. Using 300 intraoral clinical photographs of patients' gingiva, logistic regression, neural network, and naive Bayes were trained and tested for prediction and detection. Accuracy was measured using a confusion matrix and receiver operating characteristic (ROC) curve.

Results

In the present study, three convolutional neural network (CNN)-embedded machine-learning algorithms detected and predicted anaemia. For anaemia identification, naive Bayes had an area under curve (AUC) of 0.77, random forest plot had an AUV of 0.78, and logistic regression had 0.85. Thus, the three machine learning methods detected anaemia with 77%, 78%, and 85% accuracy, respectively.

Conclusion

Using artificial intelligence (AI) with clinical intraoral gingiva images can accurately predict and detect anaemia. These findings need to be confirmed with larger samples and additional imaging modalities.

## Introduction

A person with anaemia has a haemoglobin concentration two standard deviations below the usual range for their age and gender, in men aged over 15 years (Hb below 130 g/L), in non-pregnant women aged over 15 years (Hb below 120 g/L), and in children aged 12-14 years of age (Hb below 120 g/L). Acute anaemia develops immediately, while chronic anaemia takes longer. Anaemia, a global public health issue, primarily impacts youngsters and pregnant women [1]. According to the World Health Organisation (WHO) studies conducted in the year 2020, iron deficiency anaemia has affected 33% of the world's population, also 40% of pregnant women and 42% of children under 15. A decrease in red blood cell production or structural damage causes anaemia [2].

Anaemia happens either when the body's red blood cell production declines or the cells' structural integrity is compromised [3]. Anaemia can also appear when the haemoglobin level in the red blood cells drops below the typical threshold as a result of increases in red blood cell oxidation, blood loss, defective cells, or a reduction in the quantity of red blood cells [1]. One of the key strategies for treating anaemia is early detection [4] and therapeutic intervention to prevent irreversible organ damage.

Symptoms include fatigue, disorientation, drowsiness, and weakness. Patients with chronic illnesses may develop anaemia [5]. In addition, anaemia has been shown to reduce adult productivity, impact children's psychological and physical development, and, when left untreated, can result in health issues like extreme fatigue and pregnancy complications. With an ongoing illness, there is also a chance that a patient will develop anaemia [6]. There are many different types of anaemia, including iron deficiency, sickle cell disease, thalassemia, aplastic anaemia, and vitamin or iron deficiency. Every type of anaemia has a wide variety of causes that can be temporary or persistent, mild to severe [2]. In susceptible hosts, certain germs induce periodontitis, an inflammatory illness that destroys tooth-supporting tissues. Periodontitis is usually caused by Gram-negative anaerobic bacteria. Systemic exposure to bacteria and their products in periodontal tissues generates a strong vascular response and an immunological inflammatory response [7].

Periodontitis-related localized infections can have a significant impact on both people's and animals' overall health. In addition to mounting an immune inflammatory response to bacteria and their products in the periodontal tissues, systemic exposure to these agents also causes a significant vascular response [7]. The sulcular epithelium protects the body against irritants and bacteria. The host-microbe interaction in periodontitis ulcerates the sulcular epithelium. The ulcerated pocket epithelium lets bacteria infiltrate connective tissue, enter the bloodstream, and produce bacteremia. Bacteremia and inflammation are linked to periodontitis [8]. The interaction between periodontitis and a number of systemic disorders may be explained by the host response. Cancer, infections, and autoimmune disorders activate the immune system and produce cytokines. The most often generated cytokines are tumor necrosis factor (TNF), interleukin (IL)-1, and IL-6 [9]. Decreased erythropoietin (EPO) synthesis by inflammatory cytokines can cause anaemia [10]. Chronic disease-related anaemia diseases are characterized by macrophage-derived inflammatory cytokines like IL-1, IL-, IL-6, transforming growth factor, and tumour necrosis factor (TNF). Cellular or humoral substances like TNF and IL-1 decrease the bone marrow's EPO response in chronic inflammatory diseases, which may cause this anaemia. Thus, periodontitis may cause modest systemic inflammation, lowering red blood cell count and haemoglobin. The link between periodontal disease and anaemia is disputed.

Insufficient funding for medical tests, a lack of technical expertise and equipment in remote areas, requirements for quality, and client resistance that leads to abstinence are just a few of the difficulties faced in practice by the laboratory procedure for the diagnosis and detection of anaemia in response to clinical concerns [11]. Invasive procedures put healthcare personnel at risk of bloodborne diseases [12]. Non-invasive anaemia detection methods like machine learning algorithms have gained popularity to solve these challenges.

Recent clinical procedures for diagnosing anaemia require blood extraction, which is laborious, financially straining, and time-consuming [13]. Many hospitals identify anaemia rapidly by examining the conjunctiva. Medical specialists pull down the eye's protective fold and subjectively evaluate the fingernails, palm, and conjunctiva. However, the variability of inter-observer agreement in various places, the decreased sensitivity of the conjunctiva colour, and the palpable palm will invalidate the physical examination technique. Clinical signs for anaemia identification can change and be helpful. Without expensive and sometimes unavailable blood testing, this scenario could improve. Thus, early anaemia detection is challenging [13].

Non-invasive methods and smartphone-based gadgets can diagnose and monitor anaemia risk. This paper evaluates and investigates trends and theories on machine learning in healthcare and finds methods for reliable anaemia diagnosis using medical photos. We also compare the most successful algorithms and evaluate the model that performs better in that study. As they are affordable, simple, and non-invasive, machine-learning methods for detecting anaemia have been proposed. Most developing nations lack medical staff, which has boosted demand for technology, especially innovative artificial intelligence (AI) models. 

Several machine learning techniques for the detection of anaemia have been proposed because they are affordable, simple to use, and non-invasive in nature [14]. The health sector has witnessed significant growth in technological advancement over the past 10 to 15 years, and scientists studying AI have been attempting to understand human language since the 1950s [15]. 

## Materials and methods

The Ethical Committee of Saveetha Dental College and Hospitals (Chennai, India) approved this study (approval no. IHEC/SDC/PERIO-2105/23/074). The intraoral clinical pictures of patients’ gingiva used in this study were selected from a dental information management software database. Three hundred images were acquired from Saveetha Dental College and Hospitals and annotated and segmented for further processing. Experts’ manual labelling provided a reference for training and evaluating the models.

The study was carried out using the Orange Data Mining tool (developed by the Bioinformatics Lab at the University of Ljubljana, Slovenia) [16]. The machine learning approach uses image mining with visual analytics leading to automatic data interactive visualisations. Orange enables file loading, transformation, visualisation with user interaction, model inference, and model visualisation, as well as all of the critical steps required for visual analytic frameworks. Images from the penultimate layer of multiple deep image classification models from the Keras Python library, including InceptionV3, squeeze net, painters, and deep loc, are shown in orange.

To obtain the vector representation of a picture, embedding sends it via an existing deep network. Different embedders can be used with the Image Embedder widget in Orange.

SqueezeNet is a deep learning model known for its lightweight architecture, designed to achieve high accuracy with a significantly reduced number of parameters. SqueezeNet achieves parameter reduction by utilising various techniques, including 1x1 convolutions and fire modules that contain both squeeze and expand layers. The squeeze layers use 1x1 convolutions to decrease the number of channels, while the expand layers increase the number of channels using a combination of 1x1 and 3x3 convolutions. This efficient architecture allows SqueezeNet to achieve accuracy comparable to larger models with a significantly smaller memory footprint. Its lightweight nature makes it particularly useful for scenarios where computational resources are limited without compromising performance. To acquire findings, data were divided between 80% training and 20% testing, with 20 models using logistic regression, random forest, and naive Bayes widgets.

The procedures employ multidimensional scaling, model scoring, and cross-validation. Orange incorporates standard Python libraries for machine learning and data manipulation, such as NumPy, scipy, and sci-kit-learn, and wraps their functionality within workflow building blocks that provide an interface for changing machine-learning method parameters or browsing results and related visualisations of inferred models.

## Results

Our study involved the recruitment of 345 patients, with corresponding pictures taken of their gingiva. Two experienced periodontists were tasked with evaluating the picture quality, leading to the removal of 45 pictures due to either low definition or incomplete display of gingival images. Consequently, 300 gingival pictures representing 300 patients were utilized in the research. Among these, 210 patients were diagnosed with anemia based on complete blood count results, with an average hemoglobin concentration of 10.55g/dl.

Orange data mining is a data analysis platform that includes clustering, classification, and interactive data and model vizualisation components. The picture-specific enhancements mentioned in this research are included in Orange's image analytics add-on. Orange and its extensions are free and open source.

**Figure 1:**
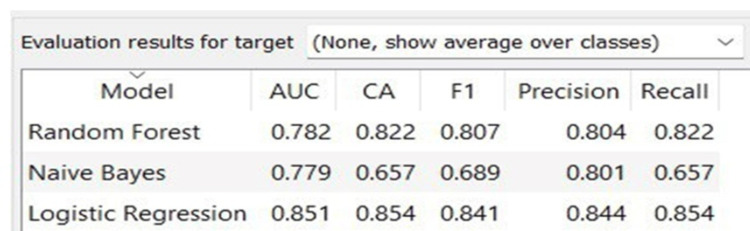
Accuracy of various algorithms used for anaemia detection AUC = area under the curve

Data are fed into widgets, which then display or convey the findings. Widget selection and connections define orange data analysis pipelines. Loading images from the specified directory, embedding them with feature vectors, calculating the distances between these vectors and thus between the photos, and then grouping and visualising image similarities in the multidimensional scaling plot using the obtained distances. The accuracy of the different algorithms employed is depicted in Figure 1. 

**Figure 2:**
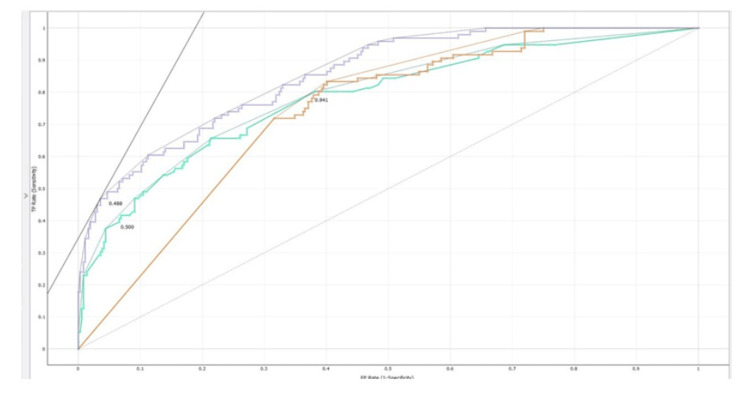
Receiver operating characteristics (ROC) for anaemia detection

The receiver operating characteristics (ROC) curve for anaemia detection is depicted in Figure 2. 

**Figure 3:**
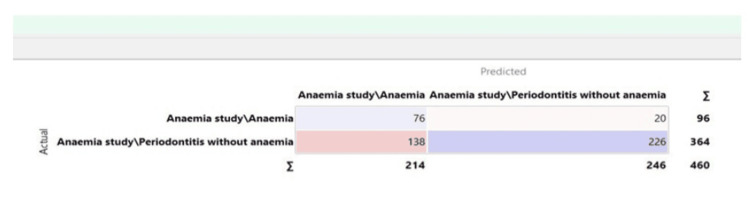
Confusion matrix for the random forest model

The confusion matrix for the random forest model is depicted in Figure 3.

**Figure 4:**
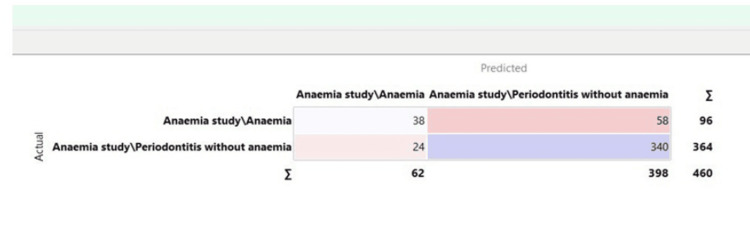
Confusion matrix for the naive Bayes model

The confusion matrix for the naive Bayes model is depicted in Figure 4. 

**Figure 5:**
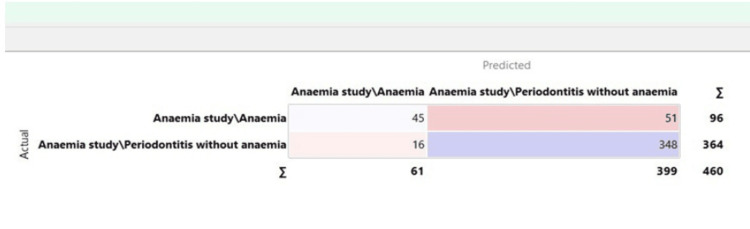
Confusion matrix for the logistic regression model

The confusion matrix for the logistic regression model is depicted in Figure 5.

Confusion matrix

It provides a comprehensive summary of the predicted and actual values of the target variable. The matrix typically comprises four cells that represent different combinations. Each cell represents the number of instances classified under that specific combination. The confusion matrix helps identify misclassification patterns and provides insights into the model's strengths and weaknesses. Overall, it is a useful tool for understanding the performance of a classification model and making informed decisions about its effectiveness.

ROC curve

The curve is created by plotting the true positive rate against the false positive rate for different thresholds, resulting in a smooth curve that ranges from 0 to 1. The area under the ROC curve (AUC-ROC) is often used as a single metric to measure the overall predictive power of the model. A higher AUC-ROC value indicates better model performance in distinguishing positive and negative instances. The ROC curve provides visualization and quantification of the model's discrimination ability, allowing comparisons between different models and aiding in decision-making regarding the model's effectiveness. Accuracy evaluates a model's dataset prediction accuracy using the f1 score. Logistic regression outperformed naïve Bayes and neural networks in the F1 score. A good classifier should have one precision (high). Precision is 1 when true positive (TP) = TP + false positive (FP), where the numerator and denominator are identical. Recall is 1 when TP = TP + false negative (FN), meaning FN is zero. As FN increases, the denominator becomes more essential than the numerator, lowering memory. FP and FN should be zero in a good classifier because accuracy and recall should be 1. F1-score, a metric combining accuracy and recall, is calculated as: [(Precision x Recall) / (Precision + Recall) x 2]. AUC-ROC curves, recall, precision, specificity, and accuracy are calculated using confusion matrices.

## Discussion

The WHO estimates that over two billion people worldwide suffer from anaemia [17]. Rapid anaemia screening and diagnosis are becoming more popular. Theoretically, anaemia may cause pallor in fingernail beds, conjunctiva, palmar wrinkles, and other areas.

In data-driven machine learning, computers learn and predict. Machine learning classifies photographs by detecting objects, people, and other attributes. Picture classification uses supervised, unsupervised, and deep learning. Decision trees, k-nearest neighbours, and neural networks improve picture categorization accuracy, efficiency, and robustness. Image machine learning is a fast-growing field that teaches computers visual information. Large photo datasets train algorithms to spot patterns, objects, and complex relationships in images. These methods apply to image categorization, object detection, segmentation, and creation.

Recent studies show that fingernail beds and conjunctiva can detect anaemia. Lack of melanocytes affects haemoglobin's red light reflection, helping diagnose anaemia. Since patients must move to disclose their conjunctiva or fingernail beds, researchers need patient participation. This movement may be easy for healthy people or those with minor illnesses, but extremely anaemic or coma patients may struggle, impacting research conclusions due to inadequate exposure to these characteristic locations. In prior investigations, some data were eliminated after filtering because feature areas were not fully exposed, lowering data availability [18].

The study employed Orange, an open-source software package designed for image analytics, which is built on top of the Orange visual programming data mining framework. Orange offers a range of functionalities, such as the construction of workflows, data modelling, interactive visualizations, clustering, classification, regression, outlier detection, and dimensionality reduction. This study used Orange to analyze small image sets using trained deep networks and image embedding. The Orange embeddings are in deep networks, in the penultimate layer. Transfer learning was made possible by encoding images with features from this layer and using machine-learning methods such as random forests or logistic regression. This approach provided a laptop-compatible and efficient solution that does not require retraining the deep neural network embedding. Orange aims to provide an interactive and accessible environment for image analytics with high functionality adaptable to specific needs. The tool can complement other toolboxes and provide an alternative to advanced users or data scientists.

Neural networks, logistic regression, and naive Bayes were tested for their ability to automatically detect and classify anaemia using intraoral clinical photographs of patients' gingiva. AI-based gingiva anaemia prediction from clinical images is novel. Anaemia can cause oral symptoms like gum and mucosal pallor due to low blood oxygen. AI models are utilized in medical imaging to diagnose. Due to the complexity of gum discolouration factors, applying them to predict anaemia from gingival imaging may be difficult.

Recently, AI and machine learning have enabled picture analysis to find patterns or indicators linked with certain illnesses. These patterns could be learned by an AI model trained on gingival pictures with anaemia diagnoses. Colour, texture, and potentially other oral indications of anaemia may be considered. Gingival scans may not accurately indicate anaemia due to gum discolouration's multifactorial character. Gum appearance can alter due to anaemia and other medical disorders. Blood tests and clinical assessments are usually needed to diagnose anaemia. This AI-based non-invasive technique enables us to accurately detect anaemia presence and correlate it with periodontitis using clinical intraoral pictures of the gingiva, as opposed to the invasive bloodwork needed when a patient has to be diagnosed as an anaemic.

Limitations

The study was conducted using clinical intraoral pictures of patients; hence, there will be heterogeneity in the quality of images. For the validation of an anaemia diagnostic technique, the use of a gold standard method is indispensable. The generalizability of the results could also be impacted by the study's relatively small sample size. The validation set was sourced from the same hospital, and conducting multicenter validation is a crucial objective for future studies. As our research participants were exclusively a South Indian population, the findings may not be directly applicable to other individuals. Examining the less-than-perfect outcomes of our study, particularly concerning mild and severe anaemia, we attribute the limitations to two factors: minimal gingival changes in mild anaemia cases and inadequate data. Nonetheless, the positive outcomes of this study persuade us that conducting further research with a larger dataset will yield improved results.

## Conclusions

Machine learning algorithms can accurately detect the presence of anaemia with the use of clinical intraoral pictures. Using larger sample sizes and different imaging modalities, additional research is required to confirm these findings. The clinical application of machine learning algorithms in dental practice should also be the main topic of future research. This holds significant clinical value and practical importance as it speeds up diagnoses, enhances the allocation of medical resources, and sets the stage for appropriate treatment in the future.
